# Characterizing the Long Non-Coding RNA Profile of Endometrial Mesenchymal Stem/Stromal Cell-Derived Extracellular Vesicles and Their Anti-Inflammatory Role in Osteoarthritis

**DOI:** 10.3390/ijms262110567

**Published:** 2025-10-30

**Authors:** Cole Conforti, Darden Wood Kimbrough, Neep Patel, Michelle B. R. G. Ley, Jose Medina Flores, Diego Correa, Lee D. Kaplan, Thomas M. Best, Dimitrios Kouroupis

**Affiliations:** 1 Department of Orthopaedics, UHealth Sports Medicine Institute, Miller School of Medicine, University of Miami, Miami, FL 33146, USA; cmc732@med.miami.edu (C.C.); dwk45@med.miami.edu (D.W.K.); ncp87@med.miami.edu (N.P.); ley.michelle@miami.edu (M.B.R.G.L.); diecorr1@gmail.com (D.C.); kaplan@med.miami.edu (L.D.K.); txb440@med.miami.edu (T.M.B.); 2 Diabetes Research Institute & Cell Transplant Center, Miller School of Medicine, University of Miami, Miami, FL 33136, USA; 3 Department of Biomedical Engineering, University of Miami, Miami, FL 33146, USA; 4 CryoVida Biotech, Guadalajara 44500, Mexico; presidencia@neorigen.com

**Keywords:** mesenchymal stem/stromal cells, extracellular vesicles, long non-coding RNA, inflammation, osteoarthritis

## Abstract

Endometrial tissue-derived mesenchymal stem/stromal cells (eMSCs) have potential therapeutic properties partially exerted via their secreted extracellular vesicles (EVs). eMSC-EVs contain cargos with regenerative and immunomodulatory properties. Specifically, the miRNA profile of CD146High eMSC-EVs has been shown to promote anti-inflammatory M2 macrophage polarization in vitro. Herein, we aimed to characterize the lncRNA profile of CD146High and CD146Low eMSC-EVs and further assess their immunomodulatory and anabolic therapeutic function in osteoarthritis (OA). We hypothesized that the CD146High eMSC-EVs lncRNA profile is enriched with potent anti-inflammatory and pro-anabolic cartilage effects when compared to the CD146Low eMSC-EVs lncRNA profile. Human endometrial tissue was collected, and the eMSCs were magnetically sorted to yield the CD146High and CD146Low eMSC subpopulations. The eMSC-EVs were isolated via ultracentrifugation and CD63 magnetic immunoselection methods and characterized by nanosight and flow cytometry analyses. Our results showed that CD146High eMSC-EVs display an lncRNA profile with both anabolic and catabolic features, exerting a more dynamic effect on chondrocyte gene expression than CD146Low eMSC-EVs, suggesting a potential benefit of using CD146High eMSC-EVs to attenuate the negative effects of inflammation in OA. CD146High eMSC-EVs also demonstrated greater endothelial repair capacity under inflammatory stress. In conclusion, cell-free CD146High eMSC-EV has therapeutic potential through its protective anti-inflammatory effects, warranting further pre-clinical investigation.

## 1. Introduction

Endometrial tissue-derived mesenchymal stem cells (eMSCs) are a dynamic population of cells that exhibit immense regenerative and immunomodulatory capacities, making them a strong candidate for stem cell-based therapies. First characterized in 2004 by Chan et al., eMSCs are localized to the basal layer of the endometrium and are responsible for the monthly regeneration of the endometrial functional layer [1,2,3]. These cells are easily extractable and exhibit multilineage-differentiation potential in vitro [4,5]. eMSCs are further classified into epithelial-like, stromal-like, or perivascular subgroups, with each containing a distinct molecular and functional profile [6]. The perivascular subgroup is a CD146+/CD140b+ population that displays high clonogenic capacity when isolated in vitro [1,7]. Similarly to the endometrial subgroup, perivascular MSCs throughout the body are marked by CD146 expression [8]. Functionally, these cells can detect systemic and local inflammation, migrate to the source, and secrete extracellular vesicles (EV) to dampen inflammation and promote tissue repair. In our prior work, we have demonstrated that CD146+ bone marrow-derived MSCs show markedly higher secretory capacity with significantly greater immunomodulatory and anti-inflammatory protein production upon inflammatory priming compared to CD146− MSCs [9]. Specifically, the study demonstrated that CD146+ MSCs more effectively attenuate PBMC inflammatory secretions and induce Treg phenotypes in vitro, whereas promoting M2 macrophage polarization in vivo by ameliorating inflammation/fibrosis of the knee synovium and fat pad. Likewise, initial studies of the eMSC CD146+ perivascular subgroup show this population to have enhanced immunomodulatory and angiogenic properties [10,11]. Given these features of CD146+ eMSCs—namely, the dynamic nature of their source tissue and their ease of extractability—CD146+ eMSCs offer clinical benefits over MSCs derived from other anatomical compartments such as the bone marrow or adipose tissues. Thus, further research characterizing the mechanisms of immunomodulation and angiogenesis of eMSCs is necessary to discern their therapeutic efficacy.

The therapeutic potential of eMSCs is mainly extended through their EVs, which are non-immunogenic secreted vesicles that function by transporting bioactive molecules, including nucleic acids (DNA, RNA, miRNA, and lncRNA), proteins, lipids, and metabolites, from a parental cell to target cells [11,12,13,14]. MSC-derived EVs (MSC-EV) have demonstrated therapeutic efficacy in clinical trials of numerous diseases, such as neurologic diseases, diabetes, wound healing, and COVID-19 [15]. Despite EVs gaining popularity in research due to their therapeutic potential and ability to deliver beneficial cargos, there are few studies on EVs derived from eMSCs (eMSC-EVs) [12,16]. Although limited, studies suggest eMSC-EVs exert immunomodulatory and pro-angiogenic effects both in vitro and in vivo [11,17,18,19,20]. In our previous study, we demonstrated that CD146+ eMSC-EVs possess a more favorable anti-inflammatory miRNA profile compared to EVs from an unselected eMSC population, and that they functionally modulate macrophage polarization and peripheral blood mononuclear cell (PBMC) activity under inflammatory conditions in vitro [21]. Additionally, the pro-angiogenic properties of eMSCs via secretory products [11] are hypothesized to be related to the contents of eMSC-EVs. To better understand the anti-inflammatory mechanisms of eMSC-EVs, additional EV-associated molecular components should be explored. Long non-coding RNAs (lncRNAs), in particular, may interact with the miRNA cargo and help explain the therapeutic effects of these vesicles.

LncRNAs are defined as RNA transcripts greater than 200 nucleotides long that lack protein-coding capabilities [22], and they are a key component of eMSC-EVs because of their interactions with miRNAs, DNA, and proteins in target cells to modulate various cellular processes. Specifically, lncRNAs are important modulators of the immune response. For example, lincRNA-EPS has been shown to modulate expression of immune response genes in macrophages through chromatin remodeling and transcription repression [23]. Additionally, lncRNA imbalances have been identified in various disease states, including osteoarthritis (OA). Compared to healthy controls, OA patients have lower levels of lncRNA TUC339, a lncRNA derived from bone marrow MSCs with anti-inflammatory and pro-chondrogenic properties [24].

OA is the most common joint disease, affecting millions worldwide, that is now understood as a heterogeneous condition driven by chronic inflammation, altered biomechanics, and metabolic dysfunction [25]. While the initial development of OA is not always driven by systemic inflammation, damage to the cartilage, synovium, and subchondral bone is maintained and progressed by both innate and adaptive immune cells [26]. We hypothesize that eMSC-EVs have the potential to dampen these processes in OA and induce restorative, anabolic effects in affected tissues.

In the present study, therapeutic lncRNA profiles of EVs isolated from CD146High and CD146Low eMSC populations were characterized and compared. Additionally, we investigated the capacity of eMSC-EVs to affect chondrocyte and endothelial cell functionality under inflammatory conditions. Characterization of the lncRNA content of CD146High eMSC-EVs will elucidate the mechanisms underlying their therapeutic effects, reveal pathways associated with their known miRNA cargo, and support further investigation of these EVs as cell-free therapeutics for chronic inflammatory conditions like OA.

## 2. Results and Discussion

### 2.1. CD146High and CD146Low eMSC Characterization

**Figure 1:**
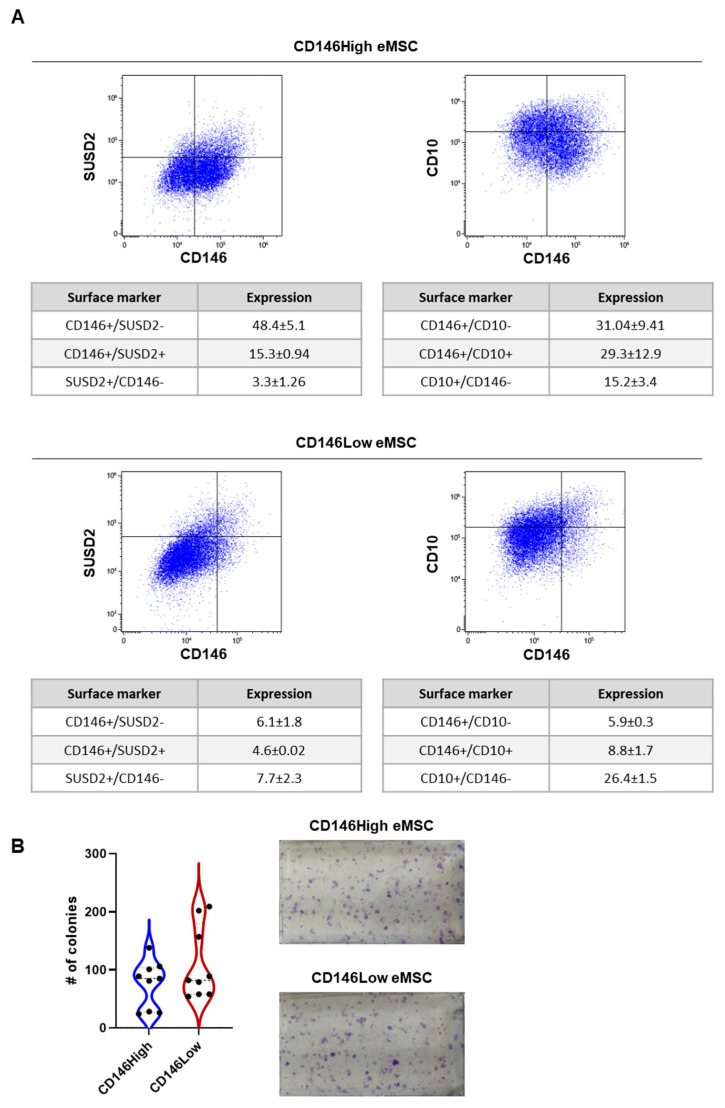
CD146High and CD146Low eMSC immunophenotype and clonogenicity. (**A**) CD146 eMSCs show higher coexpression of CD146 with SUSD2 and CD10; (**B**) CD146High and CD146Low eMSCs show similar clonogenicity.

We first isolated CD146High and CD146Low eMSCs through an immuno-magnetic separation technique [11]. CD146High and CD146Low eMSCs were expanded until P4, and the expression of CD146, SUSD2, and CD10 was evaluated. CD146High eMSCs demonstrated CD146+/SUSD2+ co expression of 15.3% +/− 0.94 and CD146+/CD10+ co expression of 29.3% +/− 12.9. CD146Low eMSCs demonstrated CD146+/SUSD2+ co expression of 4.6% +/− 0.02 and CD146+/CD10+ co expression of 8.8% +/− 1.7 (Figure 1A). After confirming positive and negative selection of the CD146High and CD146Low eMSCs, respectively, we assessed the functional capacities of each subgroup. As expected, both subgroups exhibited similar clonogenic capacities, demonstrating that each retained stemness after positive or negative selection, respectively (Figure 1B).

### 2.2. CD146High and CD146Low eMSC-EVs Possess Immunomodulatory/Angiogenic lncRNA Cargo

Isolation and characterization of eMSC-EVs was performed following the techniques described in our prior study [11]. Specifically, characterization demonstrated efficient isolation of EVs with >90% purity (CD63+ CD9+ expression) and <200 nm size assessed by flow cytometry and nanoparticle tracking analysis, respectively [11].

**Figure 2:**
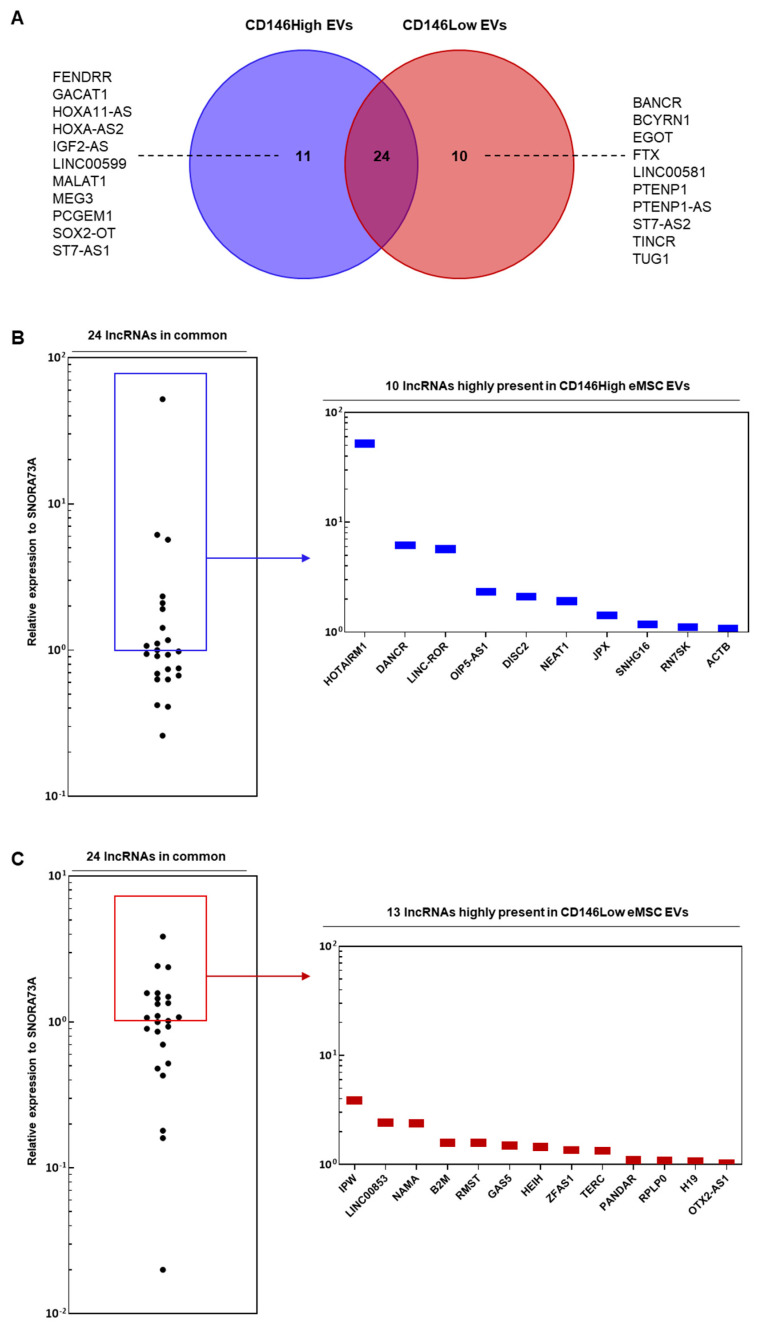
(**A**) Most lncRNAs were commonly expressed in the eMSC-EVs; however, 11 lncRNAs were uniquely present in CD146High eMSC-EVs (FENDRR, GACAT1, HOXA11-AS, HOXA-AS2, IGF2-AS, LINC00599, MALAT1, MEG3, PCGEM1, SOX2-OT, ST7-AS1) and 10 lncRNAs were uniquely present in CD146Low eMSC-EVs (BANCR, BCYRN1, EGOT, FTX, LINC00581, PTENP1, PTENP1-AS, ST7-AS2, TINCR, TUG1); (**B**) 10 of the 24 lncRNAs commonly expressed by both groups had a higher presence in CD146High EVs (HOTAIRM1, DANCR, LINC-ROR, OIP5-AS1, DISC2, NEAT1, JPX, SNHG16, RN7SK, ACTB); (**C**) 13 of the 24 lncRNAs commonly expressed by both groups had a higher presence in CD146Low EVs (IPW, LINC00853, NAMA, B2M, RMST, GAS5, HEIH, ZFAS1, TERC, PANDAR, RPLP0, H19, OTX2-AS1).

**Figure 3:**
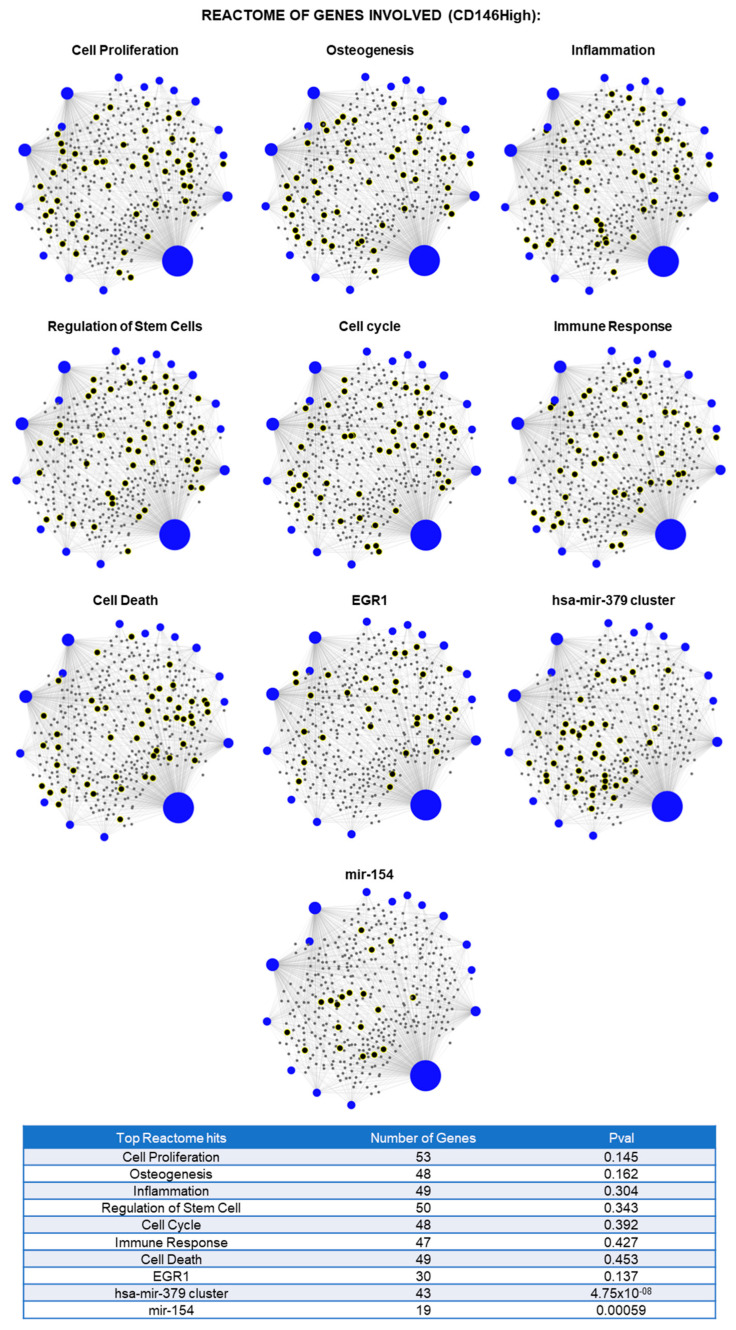
Reactome analysis of 21 lncRNAs uniquely or highly present in CD146High eMSC-EVs showed their involvement in the regulation of ten gene or miRNA groups related to cell proliferation, osteogenesis, inflammation, regulation of stem cells, cell cycle, immune response, cell death, EGR1, hsa-mir-379 cluster, and mir-154 (blue spots: lncRNA detected in CD146High eMSC-EVs, gray spots: genes related to pathways, black spots with yellow highlight: genes related to the pathway that are affected by lncRNAs detected).

From the lncRNAs investigated, there were 45 total lncRNAs present in CD146High EVs and CD146Low EVs (Figure 2). Of these, 11 were uniquely present in the CD146High population and 10 were uniquely present in the CD146Low population (Figure 2A). There were 24 lncRNAs shared between both groups. Next, we sought to determine which of the 24 common lncRNAs were enriched in either population.-fold change analysis demonstrated 10 lncRNAs enriched in the CD146High population (>100 expression relative to SNORA73A) and 13 lncRNAs enriched in the CD146Low group (Figure 2B,C). Furthermore, among the 21 uniquely present or enriched lncRNAs in the CD146High population, miRNet pathway analysis predicted the lncRNAs to be involved in cellular processes such as cell proliferation, osteogenesis, inflammation, and regulation of stem cells (Figure 3). These findings suggest a potential role for these lncRNAs in regulating the anabolic/catabolic balance and inflammatory response in arthritic joint spaces.

Some of the lncRNAs solely present or enriched in the CD146High EVs were HOXA-AS2, IGF2-AS, PCGEM1, MALAT1, MEG3, HOTAIRM1, FENDRR, DANCR, LINC-ROR, SOX2-OT, ST7-AS1, HOXA11-AS, OIP5-AS1, DISC2, and LINC00599 (Figure 2A,B). These lncRNAs comprise a diverse set of genes that possess a wide range of context-dependent functions throughout the body. Given the vast set of cellular processes that lncRNAs coordinate, we directed our focus to the anabolic/catabolic and immunomodulatory effects of the lncRNAs to discern their potential therapeutic role in treating OA.

Firstly, the enriched lncRNAs in the CD146High EVs that have demonstrated anabolic and anti-inflammatory roles include HOXA-AS2, IGF2-AS, MEG3, DANCR, and OIP5-AS1 (Figure 2A,B). HOXA-AS2, IGF2-AS, and MEG3 were uniquely present in CD146High EVs. HOXA-AS2 inhibits NF-kB signaling, which is well-known to promote cartilage catabolism in OA [27,28]. Additionally, HOXA-AS2 upregulates SMAD2 [29], which promotes TGF-β signaling, a crucial pathway for maintaining cartilage integrity in OA [30,31]. Similarly, IGF2-AS exhibits anabolic features through promoting osteogenesis in BM-MSCs [32] and myogenesis in bovine myoblasts [33]. MEG3 has demonstrated anti-OA effects in different OA models. Studies show that MEG3 down-regulates ECM degradation, represses inflammation and exhibits chondro-anabolic effects by functioning through different miRNA axes [34,35,36,37]. Lastly, DANCR and OIP5-AS1 up-regulated 6.1- and 2.3-fold, respectively, in CD146High EVs, have demonstrated pro-anabolic and anti-inflammatory features in an OA context [38]. The significant enrichment of these pro-anabolic, anti-inflammatory biomolecules in the CD146High EVs suggests a potential therapeutic benefit for their use in OA.

However, there were a few lncRNAs solely present (Figure 2A, blue region) or enriched (Figure 2B, blue bars) in the CD146High EVs that have demonstrated more OA-catabolic effects in the literature, which include PCGEM1, MALAT1, and HOTAIRM1. Firstly, PCGEM1, solely present in CD146High EVs, functions through different miRNA axes to influence OA pathogenesis. Through downregulating miR-770, PCGEM1 increases proliferation and decreases apoptosis of synoviocytes [39]. Additionally, PCGEM1 exhibits anti-apoptotic and catabolic ECM effects through the miR-142-5p/RUNX2 axis [40]. MALAT1, solely present in CD146High EVs, can promote activation of inflammatory pathways such as NFkB and TLR4 and increase production of cytokines such as IL-1β and TNF-α [41,42,43,44]. Also, it has been shown to influence ADAMTS5 expression, which promotes ECM degradation [41]. Lastly, HOTAIRM1, upregulated 50-fold in CD146High EVs, has been shown to have pro-inflammatory and ECM-catabolic effects, with the latter functioning through upregulation of ADAMTS5 [45,46]. Although these pro-catabolic lncRNAs were enriched in the CD146High EVs, they are not as highly expressed as the pro-anabolic, anti-inflammatory lncRNAs of HOXA-AS2 and IGF2-AS. Indeed, the highest expressed “pro-catabolic” lncRNA PCGEM1 is 140-times less expressed than HOXA-AS2 and 17-times less expressed than IGF2-AS. This suggests that the anabolic lncRNAs could overshadow the catabolic lncRNAs, leading to a potential net benefit in the context of OA therapy. Nonetheless, the interaction between these different anabolic and catabolic lncRNAs warrants attention, and discerning the mechanisms underlying their overall functions in chondrocytes, synoviocytes, and osteocytes is an exciting avenue of further exploration.

**Figure 4:**
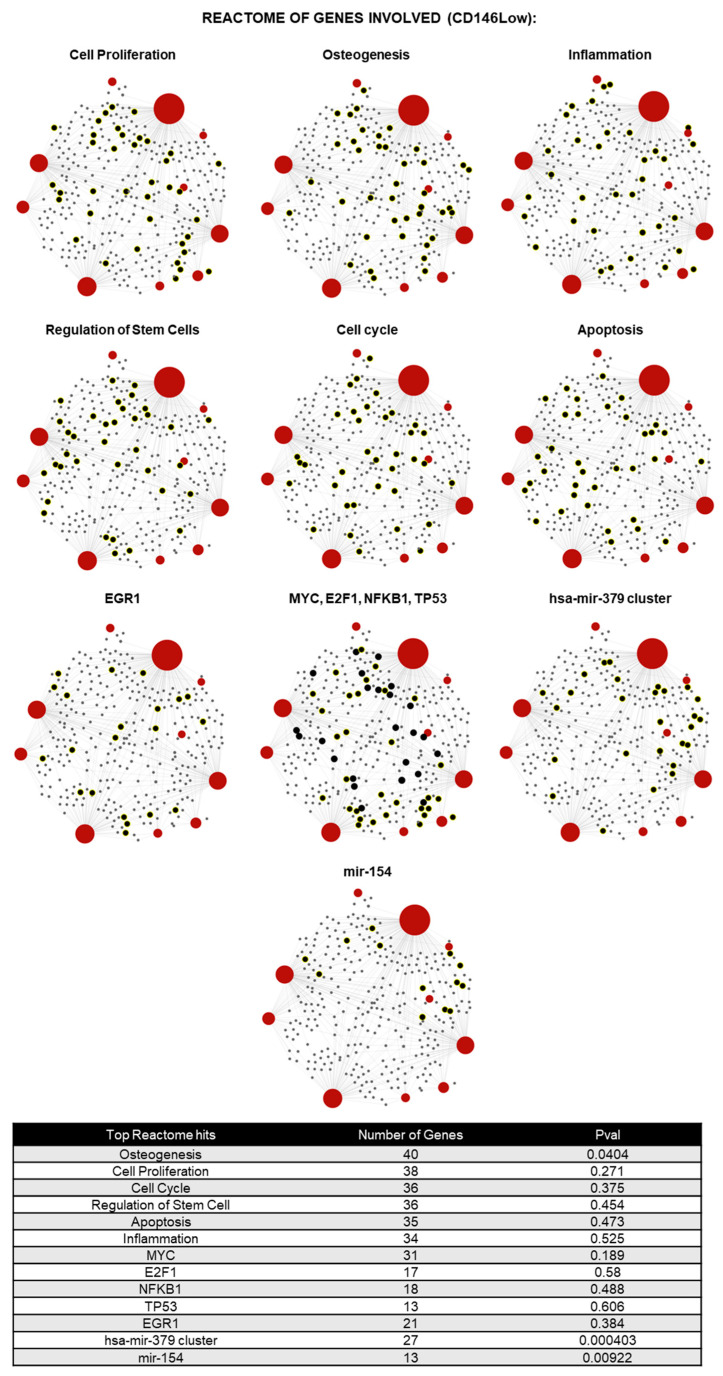
Reactome analysis of 23 lncRNAs unquely or highly present in CD146Low eMSC-EVs showed their involvement in the regulation of ten gene or miRNA groups related to cell proliferation, osteogenesis, inflammation, regulation of stem cells, cell cycle, apoptosis, EGR1, MYC, E2F1, NFKB1, TP53, hsa-mir-379 cluster, and mir-154 (red spots: lncRNA detected in CD146Low eMSC-EVs, gray spots: genes related to pathways, black spots with yellow highlight: genes related to the pathway that are affected by lncRNAs detected).

In terms of the CD146Low EVs, the 10 unique lncRNAs that were found are BANCR, BCYRN1, EGOT, FTX, LINC00581, PTENP1, PTENP1-AS, ST7-AS2, TINCR, and TUG1 (Figure 2A). Out of the commonly shared lncRNAs, there were 13 highly expressed relative to the CD146High control EVs, 4 of which had a-fold change greater than 2.00 (IPW, EGOT, LINC00853, and NAMA) (Figure 2C). Through miRNet pathway analysis, the enriched lncRNAs in what CD146Low EVs population are predicted to be involved in processes such as osteogenesis, cell proliferation, cell cycle, regulation of stem cells, apoptosis, and inflammation (Figure 4).

Among the uniquely expressed EV lncRNAs in the CD146Low population, only a few have been investigated for their role in chondrocytes, such as PTENP1 [47] and TUG1 [48,49]. PTENP1 has been found to be upregulated in OA chondrocytes compared with controls [47]. Additionally, the anti-sense codon counterpart to PTENP1, PTENP1-AS, was also uniquely expressed in the CD146Low population, and its interaction with the sense strand may play a regulatory role promoting the development of OA. Similarly, TUG1 has been found to be overexpressed in patients with OA compared with those without OA [48]. Additionally, the upregulation of TUG1 increased MMP-13 expression and decreased miR-195 expression, whereas the opposite effects were observed when TUG1 was knocked out [48]. MMP-13, or Matrix metalloproteinase-13, promotes the development of OA as it degrades cartilage through cleaving type II collagen [50]. Notably, TUG1 expression was found to be inducible by pro-inflammatory cytokines IL-1β and TNF-α [48]. Therefore, this supports the unique expression of TUG1 in CD146Low EVs, which promotes cartilage degradation and the development of OA. Although not directly investigated in its role in chondrocytes, higher expression of FTX has been shown to limit cell differentiation and proliferation in an inflammatory setting in human dental pulp stem cells (hDPSCs) by downregulating the OCT4A protein [51]. This further supports the view that CD146Low EVs lean towards a more catabolic role through degradation and limited regeneration. There were limited findings on the remaining uniquely expressed lncRNAs.

A similar trend of limited findings in relation to OA is apparent among the 4 enriched lncRNAs in the CD146Low EV population: IPW, EGOT, LINC00853, and NAMA (Figure 2C). Currently, there is a gap in the literature regarding the roles of these lncRNAs, not only in the context of chondrocytes and OA, but more broadly as well. Once again, to evaluate their therapeutic potential in treating OA, the focus will be to investigate anabolic and catabolic effects. Starting with catabolic effects, one of the highly expressed lncRNAs from the CD146Low EV population is IPW, located on the Prader–Willi Syndrome (PWS) region on chromosome 15, and is monoallelically expressed [52]. IPW is highly expressed within normal pluripotent stem cells but is heavily downregulated in patients with PWS or maternally derived egg stem cells, and is notable for its regulatory role for the DLK1-DIO3 region [53]. When the DLK1-DIO3 region becomes dysregulated, there would be upregulation of miRNAs and Maternally Expressed Genes related to PWS [53]. DLK1 is known to promote bone loss, as seen in one study where there was a decreased bone formation rate among Dlk1-overexpressing mice and is also found to promote the production of pro-inflammatory cytokines and immune response-related factors within bone marrow MSCs [54,55]. Therefore, IPW plays a more catabolic role through its epigenetic regulation of DLK1 and could have implications beyond PWS. Another enriched lncRNA with potential catabolic effects is LINC00853. In pancreatic cancer (PC) cells, LINC00853 is overexpressed, and its silencing is found to decrease tumor growth and suppress cellular glycolysis [56]. The mechanism of action of LINC00853 is through its interaction with the glycolytic regulatory enzyme PFKFB3 [56]. Given that the silencing of LINC00853 suppresses glycolysis, it may promote this catabolic process under normal conditions through interaction with PFKFB3. NAMA is another highly expressed lncRNA with potential catabolic effects. This lncRNA is believed to play a tumor-suppressive role with its role in growth arrest and apoptosis, and its interaction with the MAP kinase pathway [57]. The remaining highly expressed lncRNA within the CD146Low EVs, EGOT, has unclear net catabolic or anabolic effects. The role of EGOT is limited to cancer, where it can either play a catabolic role in terms of breast cancer through the production of autophagosomes via the upregulation of the inositol 1,4,5-trisphosphate receptor 1 (ITPR1) or an anabolic role, where there is increased cell growth, invasion, and reduced apoptosis in colon cancer [58]. Overall, a majority of the highly expressed lncRNAs within the CD146Low EV population favored a more catabolic role.

### 2.3. Molecular Profile of Chondrocytes Exposed to CD146High or CD146Low eMSC-EVs Under Inflammatory Conditions In Vitro

**Figure 5:**
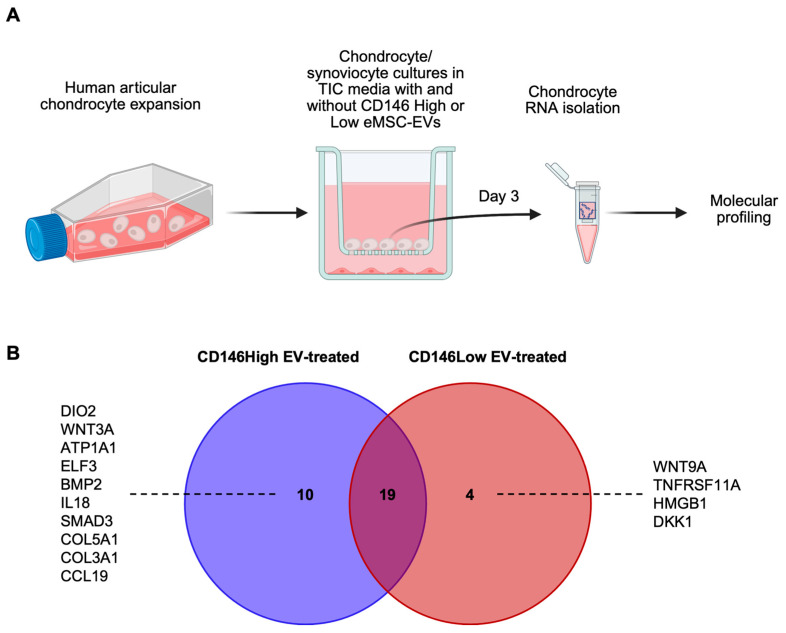
Effects of CD146High and CD146Low eMSC-EVs on chondrocytes in vitro. (**A**) Experimental strategy to simulate a pro-inflammatory environment in vitro by co-culturing human articular chondrocytes with inflamed synoviocytes; (**B**) molecular profile of chondrocytes exposed to CD146High or CD146Low eMSC-EVs under inflammatory conditions in vitro. Most genes were commonly expressed in the eMSC-EV-treated chondrocytes; however, ten genes were uniquely present in CD146High EV-treated group (DIO2, WNT3A, ATP1A1, ELF3, BMP2, IL18, SMAD3, COL5A1, COL3A1, CCL19) and four genes were uniquely present in CD146Low EV-treated group (WNT9A, TNFRSF11A, HMGB1, DKK1).

**Figure 6:**
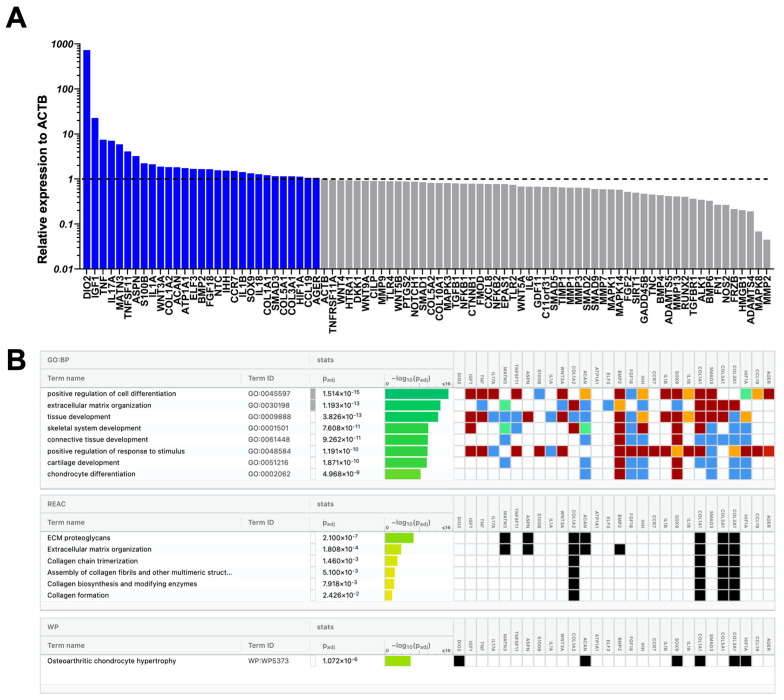
CD146High eMSC-EVs effects on chondrocyte gene expression under inflammatory conditions. (**A**) At the molecular level, treatment with CD146High eMSC-EVs increased expression of 29 genes compared to nontreated controls; (**B**) Reactome analysis of highly expressed genes in CD146High EV-treated chondrocytes showed their involvement in the regulation of 15 molecular pathways. The colors for different evidence codes and for log scale are described in Supplementary Table S1.

**Figure 7:**
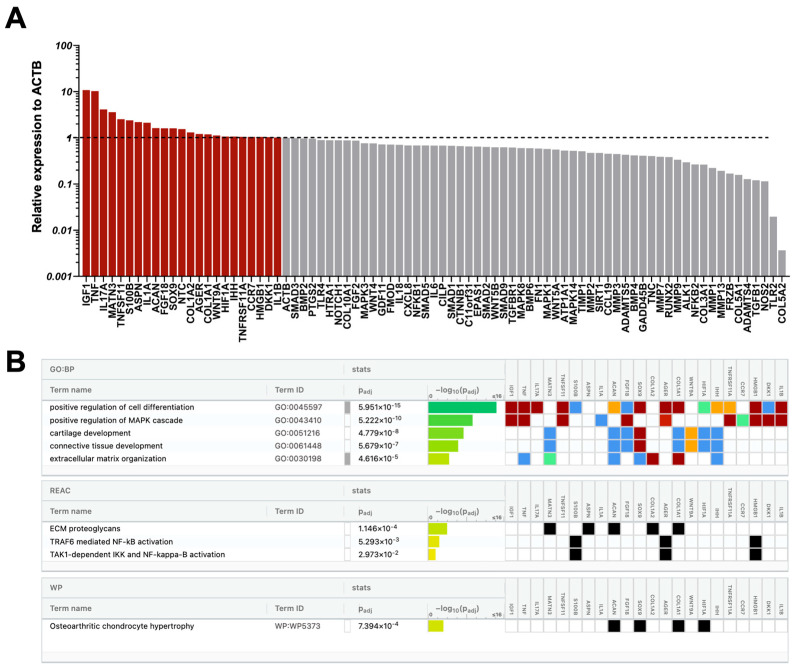
CD146Low eMSC-EVs effects on chondrocyte gene expression under inflammatory conditions. (**A**) At the molecular level, treatment with CD146Low eMSC-EVs increased expression of 23 genes compared to nontreated controls; (**B**) Reactome analysis of highly expressed genes in CD146Low EV-treated chondrocytes showed their involvement in the regulation of 9 molecular pathways. The colors for different evidence codes and for log scale are described in Supplementary Table S1.

In inflammatory joint diseases like OA, chronic low-grade inflammation disrupts the balance between catabolic and anabolic processes, resulting in maladaptive changes in articular chondrocytes [59]. Pro-inflammatory cytokines, particularly tumor necrosis factor-⍺ (TNF⍺) and interleukin-1 β (IL-1β), promote chondrocyte degradation by stimulating matrix metalloproteinases (MMPs) and inhibiting the synthesis of type-II collagen and proteoglycans, thereby contributing to both the breakdown and underproduction of extracellular matrix (ECM) [59,60]. To replicate this pro-inflammatory microenvironment in vitro, we co-cultured human articular chondrocytes with synoviocytes in cytokine-supplemented media (TNFα, IFNγ, CTGF) and treated them with eMSC-EVs from either CD146High or CD146Low subpopulations (Figure 5, Figure 6 and Figure 7).

At the molecular level, a total of 33 out of 88 genes tested showed increased expression in CD146High and CD146Low eMSC-EV-treated chondrocytes compared to non-treated chondrocytes. Of these, 19 (IGF1, TNF, IL17A, MATN3, TNFSF11, ASPN, S100B, IL1A, COL1A2, ACAN, FGF18, NTC, IHH, CCR7, IL1B, SOX9, COL1A1, HIF1A, AGER) were upregulated in both populations at varying levels (Figure 5, Figure 6 and Figure 7). 10 genes (DIO2, WNT3A, ATP1A1, ELF3, BMP2, IL18, SMAD3, COL5A1, COL3A1, CCL19) were present only in CD146High eMSC-EV-treated chondrocytes, and 4 genes (WNT9A, TNFRSF11A, HMGB1, DKK1) were present only in CD146Low eMSC-EV-treated chondrocytes (Figure 5).

Reactome analysis of the 29 highly expressed genes in CD146High eMSC-EV-treated chondrocytes showed their involvement in the regulation of 15 chondroprotective and ECM homeostasis-related molecular pathways (Figure 6B). To better understand how these pathways can be linked specifically to CD146High eMSC-EV-treated chondrocytes, we focused on the 10 genes that were uniquely expressed in this group. Two genes uniquely upregulated in CD146High eMSC-EV-treated chondrocytes are BMP2 (1.66-fold change) and SMAD3 (1.15-fold change), which have interconnected roles through the TGF-β signaling pathway. BMP2 promotes ECM formation through upregulation of type II collagen synthesis [61] and suppression of cartilage degeneration following post-traumatic OA [62]. SMAD3 plays an important role in articular chondrocyte function by supporting the expression of ECM components and suppressing hypertrophic degradation [63]. However, under inflammatory conditions like OA, post-translational inhibition of SMAD3 occurs via IL-1β and TNF-⍺, reversing these protective effects and allowing maladaptive hypertrophy due to unopposed BMP2 [64,65]. Because of this, SMAD3 upregulation alone may not be sufficient to conclude its therapeutic efficacy; however, the concurrent upregulation of ACAN (aggrecan) and SOX9, and suppression of MMP-13 and COL10A1 support its chondroprotective effect [63,64]. Another unique gene, WNT3A (1.88-fold change), regulates both canonical and non-canonical Wnt signaling pathways and contributes to cartilage differentiation and extracellular matrix (ECM) homeostasis [66]. In an in vivo mouse model of joint injury, WNT3A-loaded exosomes administered at the time of injury conferred long-term protective effects [67]. The upregulation of WNT3A observed in CD146High eMSC-EV–treated chondrocytes three days after inflammatory stimulation suggests an adaptive response, potentially mediated by the suppression of IL-1β–induced MMP13 expression (0.41-fold change) [67]. Additionally, upregulation of COL3A1 (1.15-fold change) and COL5A1 (1.15-fold change) suggests adaptive repair responses to the inflammatory stimulus. Type III collagen is synthesized in response to injury and inflammation and contributes to tissue repair and ECM stabilization [68], and type V collagen fulfills a regulatory role in the assembly of collagen fibrils [69]. Finally, the ATP1A1 gene (1.74-fold change) encodes the ⍺1-subunit of the Na+/K+ ATPase, which is essential for maintenance of the low intracellular Na+ concentration required for healthy chondrocyte function [70,71]. Thus, the increased expression of ATP1A1 in CD146High eMSC-EV–treated chondrocytes may suggest a protective role against osmotic distress.

Alternatively, some of the upregulated genes in CD146High eMSC-EV-treated chondrocytes are related to inflammatory and catabolic processes. DIO2 (deiodinase iodothyronine type 2), which was specifically upregulated only in the CD146High group with a 729.92-fold change, favors a catabolic phenotype of articular chondrocytes with reduced ECM formation and increased hypertrophy, mineralization, and MMP activity [72]. DIO2 is responsible for catalyzing the conversion of inactive thyroid hormone (T4) to active thyroid hormone (T3), favoring this catabolic state. ELF3 (1.67-fold change) and IL-18 (1.28-fold change), other genes with increased expression in this group, have parallel actions that upregulate multiple catabolic and pro-inflammatory mediators, including MMP13 and NOS2 [73,74]. However, despite the measured increased expression of DIO2, ELF3, and IL-18, CD146High eMSC-EV-treated chondrocytes displayed decreased expression of MMP13 (0.41-fold change), MMP1 (0.64-fold change), MMP2 (0.04-fold change), MMP3 (0.64-fold change), and NOS2 (0.26-fold change) compared to controls, suggesting upregulation of these genes does not translate to corresponding catabolic and pro-inflammatory effects under treatment conditions.

Reactome analysis of the 23 highly expressed genes in CD146Low eMSC-EV-treated chondrocytes also showed their involvement in the regulation of 6 chondroprotective and ECM homeostasis-related molecular pathways (Figure 7B). One gene associated with these pathways, with high expression that is unique to CD146Low eMSC-EV-treated chondrocytes, is WNT9A (1.13-fold change). In vivo mouse studies demonstrate that mice lacking WNT9A expression develop spontaneous OA, secondary to cartilage degeneration, since WNT9A is necessary for articular cartilage maintenance and lubricin expression [75,76]. Alternatively, unique to the genes upregulated in CD146Low when compared to CD146High eMSC-EV-treated chondrocytes is the involvement in 3 MAPK and NF-κB signaling pathways (Figure 7B). Interestingly, HMGB1 (high mobility group box 1) (1.05-fold change), one of four genes expressed solely in CD146Low eMSC-EV-treated chondrocytes, is involved in these pathways and contributes to maladaptive chondrocyte phenotypes in OA. HMGB1 activates the NF-κB pathway through stimulation of TLR4 and RAGE receptors, resulting in transcription of pro-inflammatory cytokines, MMPs, and pro-apoptotic molecules in chondrocytes [77,78,79]. Additionally, DKK1 (Dickkopf-related protein 1) (1.03-fold change), another gene upregulated only in CD146Low eMSC-EV-treated chondrocytes, is upregulated in response to inflammatory cytokines like IL-1β and acts via Wnt pathway inhibition, causing chondrocyte apoptosis and impaired ECM synthesis [80].

From the 19 commonly upregulated genes in both CD146High and CD146Low eMSC-EV-treated chondrocytes, IGF-1 (22.74- vs. 10.82-fold change), MATN3 (5.90- vs. 3.59-fold change), ACAN (1.81- vs. 1.62-fold change), FGF18 (1.64- vs. 1.61-fold change), and SOX9 (1.34- vs. 1.6-fold change) are involved in chondroprotective and ECM maintenance pathways. IGF-1, MATN3, FGF18, and SOX9 promote anabolic properties in chondrocytes, including ECM synthesis via type II collagen and ACAN (aggrecan) production, and inhibit apoptosis and MMPs 1–3 under inflammatory conditions [81,82,83,84,85,86,87]. Alternatively, in both CD146High and CD146Low eMSC-EV-treated chondrocyte groups, there was increased expression of TNF, IL-17A, IL-1α, IL-1β, and IHH, which have various catabolic effects. TNF, IL-17A, IL-1α, and IL-1β have catabolic and pro-inflammatory effects through an increase in MMPs, aggrecanases, and chemokines [88,89,90]. IHH (Indian Hedgehog) is upregulated in osteoarthritic articular cartilage and synovial fluid and is associated with a hypertrophic chondrocyte phenotype and increases in MMP-13 and type X collagen deposition [91]. However, despite increased expression of these catabolic genes, decreased expression of their effector genes, including various MMPs, was concurrently observed, suggesting that the transcriptome overall favors a chondroprotective phenotype.

Overall, both CD146High and CD146Low eMSC-EV-treated chondrocytes show molecular signals of both anabolic (chondroprotective) and catabolic (maladaptive) phenotypes. Reactome analysis reveals a stronger shift toward the anabolic, chondroprotective profile in CD146High eMSC-EV-treated chondrocytes.

### 2.4. CD146High and CD146Low eMSC-EVs Effect on Endothelial Cells Under Inflammatory Conditions In Vitro

Treatment with eMSC-derived EVs significantly enhanced wound closure in HUVEC monolayers compared to the untreated control. While all groups showed progressive area reduction over time, EV-treated cells exhibited faster healing under inflammatory conditions (TNFα, IFNγ, CTGF), highlighting their protective and reparative effects.

**Figure 8:**
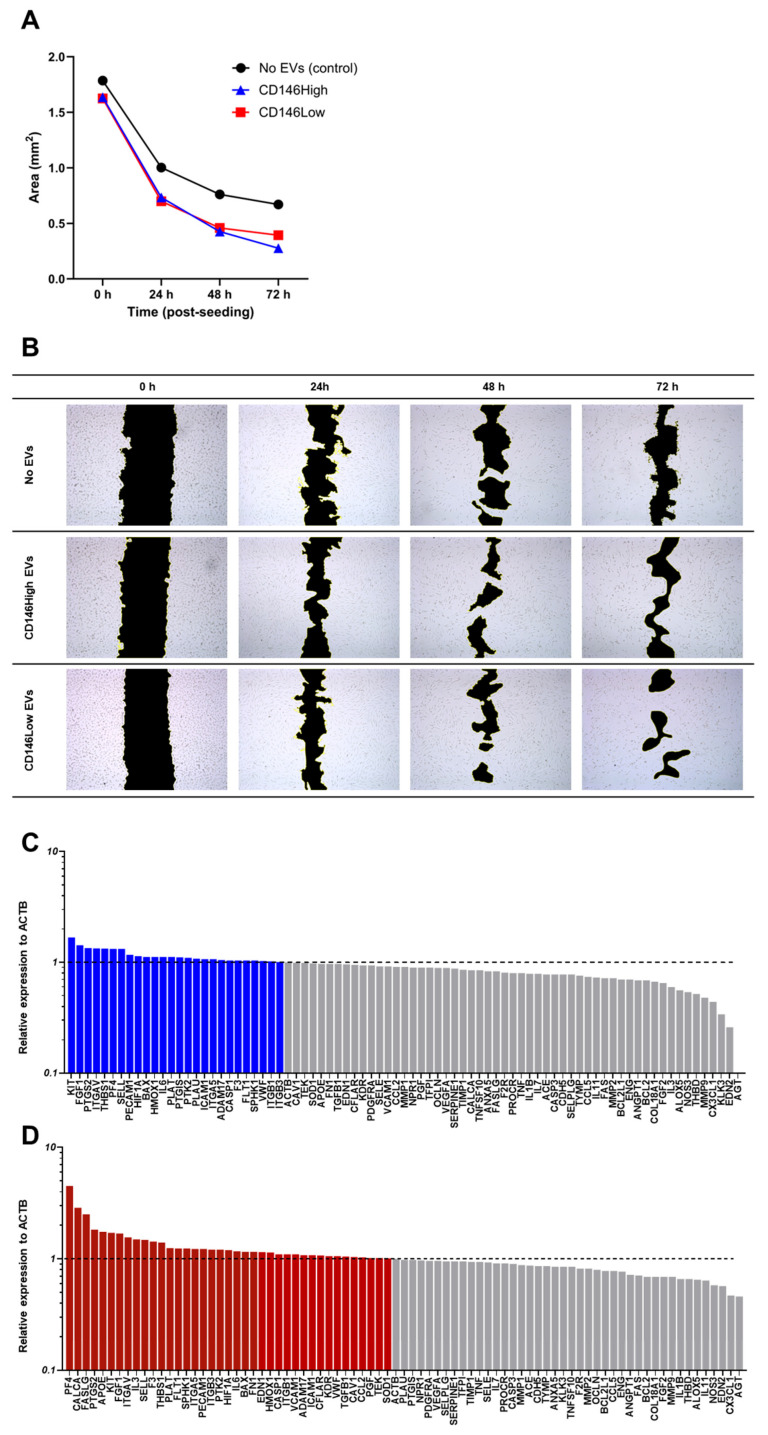
CD146High and CD146Low eMSC-EVs enhance endothelial wound closure under inflammatory conditions. Scratch assay in inflamed HUVEC monolayers demonstrates accelerated healing in cells treated with eMSC-derived EVs compared to untreated controls. (**A**) Quantification of wound area over time reveals an 83% reduction at 72 h in the CD146High group, 76% in the CD146Low group, and 62% in controls; (**B**) representative brightfield images at 0, 24, 48, and 72 h show progressive closure, with narrower wound gaps in EV-treated groups; (**C**) at the molecular level, treatment with CD146High eMSC-EVs increased expression of 26 genes in endothelial cells compared to nontreated controls; (**D**) at the molecular level, treatment with CD146Low eMSC-EVs increased expression of 39 genes in endothelial cells compared to nontreated controls.

By 24 h, the wound area was reduced by approximately 56% in the EV-treated groups, compared to 44% in the no-EV control. At 48 h, the effect of EV treatment became more pronounced, achieving approximately 73% reduction, whereas the control reached 57%. At 72 h, the CD146High group exhibited the most advanced closure (83% reduction), followed by CD146Low (76%) and the control (62%) (Figure 8A).

Representative brightfield images (Figure 8B) confirmed these quantitative findings, with enhanced closure in EV-treated wells beginning at 24 h and becoming strikingly evident by 48 h. Overall, both EV subtypes significantly promoted endothelial repair under inflammatory stress, with superior performance observed in the CD146High group.

Synthesizing the closure data with PCR analysis can further illustrate the roles of EVs. One gene expressed in endothelial cells, known for its role in angiogenesis, is AGT. Compared with the control, the CD146High group showed a fold change of 0.09, and the CD146Low group showed a fold change of 0.46. Therefore, there was a strong decrease in expression, but a much greater decrease was seen among the CD146High group. In the context of pulmonary fibrosis induced by Avian influenza viruses, higher levels of angiotensin II (Ang-II) had promoted heightened expression of TGF-β and Nuclear factor kappa B (NF-κB), as well as increased reactive oxygen species (ROS) production [92]. The catabolic signaling found with enhanced Ang-II could help explain why the greatest closure was seen with CD146High, which showed the greatest decrease in fold change relative to the control (0.09) (Figure 8C). This is then followed by the CD146Low closure rate, which also had a lower fold change, but not to the extent of CD146High relative to the control (0.46) (Figure 8A). However, it is worth mentioning that in another study that investigated the role of the AT1a (Angiotensin II Receptor Type 1a) gene, its knockout in mice resulted in suppressed wound-healing and angiogenesis [93]. Despite being a receptor for a downstream product of Angiotensinogen, it is worth considering understanding the complex role angiotensinogen and Angiotensin II have in angiogenesis.

Another gene downregulated in both groups exposed to EVs was CX3CL1. CX3CL1 is a cytokine whose receptor, CX3CR1, has been found to play a pro-angiogenic role based on findings where mice with knockout expression of CX3CR1 demonstrate delayed wound closure [94]. Interestingly, our results have found that both EV-exposed groups have had downregulated expression below a-fold change of 0.5. Therefore, there must be other characteristics within the expression profile that could explain why those groups have increased closure.

The CD146High population also had 3 other genes below a-fold change of 0.5: MMP-9 (0.48), KLK3 (0.34), and EDN2 (0.26). MMP9 is known to promote proangiogenic cytokines and induce migration of keratinocytes, thus advancing wound closure [95]. This once again may seem conflicting, given that the group with the greatest wound closure had the lowest expression of MMP9, but MMP-9 is also known to yield antiangiogenic fragments like endostatin [95]. Therefore, MMP-9 has dual opposing properties that may have yielded a net antiangiogenic effect in our experiment, but this requires further investigation. Similarly, KLK3 is another gene that has contradictory properties in terms of angiogenesis, either inhibiting angiogenic properties or promoting endothelial growth factors [96]. On the other hand, EDN2 has a clearer net antiangiogenic role, but it is limited to findings on the neural retina. The overexpression of EDN2 has obstructed endothelial cell migration and hindered vascular development of the retina in mice [97]. Although the context is different, our findings show that the group with the highest wound closure had the lowest expression of this antiangiogenic gene.

Moving on, three genes were found to have significant upregulation with a-fold change greater than 2 (PF4 (4.51), CALCA (2.88), and FASLG (2.51)) in the CD146Low EV-treated group (Figure 8D). These genes had a-fold change greater than 2 only in the CD146Low population, as there were no other genes highly expressed greater than 2 in the CD146High population. Starting with PF4 (Platelet Factor 4), which is also known as CXCL4, it is known to inhibit both angiogenesis and hematopoiesis in wound healing through binding and limiting other known angiogenesis promoters like vascular endothelial growth factor (VEGF) [98]. On the contrary, CALCA is found to have a more anti-catabolic role. CALCA or calcitonin-related polypeptide alpha encodes the calcitonin gene-related peptide (CGRP) [99]. One study found that CGRP knockout mice had reduced wound closure and angiogenesis [100]. Therefore, CGRP may have a potential pro-angiogenic role. FASLG is known for its immunosuppressive role in inducing T cell apoptosis in tumor endothelial cells [101]. It is not clear whether FASLG promotes angiogenesis, but due to its immunosuppressive role in tumor endothelial cells, FASLG has some role in regulating inflammation. In all, the genes with a-fold change greater than 2 in endothelial cells in the CD146Low population do not have a homogenous proangiogenic or anti-angiogenic role. The pathways involved are complex and overlap, requiring further investigation. Additionally, it is worth mentioning that this population had intermediate wound closure between the control with no EVs and the CD146High EVs, making the gene expression profile complex to decipher.

## 3. Materials and Methods

### 3.1. Isolation, Culture, and Expansion of eMSCs

Human endometrial tissue (n = 2) was collected according to our previous study [11] after participants provided written informed consent to the CryoVida stem cell bank (Guadalajara, Mexico). After eMSC expansion passage 0 (P0), cells were shipped to the University of Miami (Miami, FL, USA), then isolated, cultured, and expanded with complete Dulbecco’s Modified Eagle’s Medium plus 10% fetal bovine serum medium at 37 °C and 5% (*v*/*v*) CO_2_. All eMSCs were cultured by seeding 0.25 × 10^6^ cells/175 cm^2^ flask until 80% confluency until P4 and then detached with TrypLE Select Enzyme 1X (Gibco, Thermo Fisher Scientific, Waltham, MA, USA). We then assessed cell viability with 0.4% (*w*/*v*) Trypan Blue (Invitrogen, Thermo Fisher Scientific, Waltham, MA, USA). All tests were performed in accordance with the relevant guidelines and regulations following “not as human research” approval (based on the nature of the samples as discarded tissue).

### 3.2. CD146High and CD146Low eMSC Selection

The eMSC original population was sorted based on CD146 expression to yield the CD146High and CD146Low subpopulations. Briefly, crude eMSCs were re-suspended in staining buffer containing PBS with 0.5% bovine serum albumin (BSA) and 2 mM EDTA, and then incubated with biotinylated anti-human CD146 (Miltenyi Biotech, Inc., Auburn, CA, USA) at 4 °C for 20 min. The Invitrogen™ CELLection Dynabeads™ Biotin Binder Kit (Thermo Fisher Scientific) was used according to the manufacturer’s instructions for magnetic selection.

### 3.3. Immunophenotype of eMSCs

Flow cytometric analysis was performed on P4 eMSCs (n = 2). Then, 2.0 × 10^5^ cells were labeled with antibodies specific for SUSD2 (BioLegend, San Diego, CA, USA), CD146 (Miltenyi Biotec, Auburn, CA, USA), and CD10 (Biolegend) in addition to the corresponding isotype controls. All cells were stained with eFluor 780 fixable viability dye (Invitrogen). The fluorescent signal was acquired using a CytoFLEX S (20,000 events) (Beckman Coulter, Brea, CA, USA) and analyzed with Kaluza analysis software (accessed on 10 June 2025, Beckman Coulter).

### 3.4. Clonogenic Assay of eMSCSs

CD146High and CD146Low eMSCs at passage four (P4) (n = 3) were seeded in 25-cm^2^ culture flasks in triplicate, at a density of 1333 cells per plate in complete medium. Colony-forming unit fibroblasts (CFU-Fs) were manually enumerated on day 10 after cytochemical staining with 0.01% Crystal Violet (Sigma, Billerica, MA, USA).

### 3.5. Isolation and Validation of CD146High and CD146Low eMSC-EVs

CD146High and CD146Low eMSCs at passage four (P4) were seeded in complete medium until 70% confluency. Briefly, non-adherent cells were removed by Dulbecco’s phosphate-buffered saline (DPBS; Sigma Aldrich, St. Louis, MO, USA). After gentle rinsing, an exosome-depleted medium was added to each group for 48 h. Conditioned media from each group cultured in an exosome-depleted medium were collected and centrifuged at 2000× *g* for 10 min to remove debris, then at 4000× *g* for 40 min using a centrifugal filtration device (Pall Corporation, Port Wanshington, NY, USA) and finally ultracentrifuged at 120,000× *g* for 4 h. The EVs were resuspended in PBS.

### 3.6. lncRNA Profile of CD146High and CD146Low eMSC-EVs

A total Exosome RNA and Protein Isolation Kit (Thermo Fisher Scientific) was used to extract RNA from CD146High and CD146Low eMSC-EVs, according to the manufacturer’s instructions. Total exosome RNA (1 μg) was used for first-strand cDNA synthesis with the All-in-One First-Strand cDNA Synthesis Kit (GeneCopoeia, Rockville, MD, USA).

Pre-designed qPCR arrays covering 92 lncRNA primers related to human MSC exosomes (Qiagen, Hilden, Germany) were performed using 1000 ng cDNA per CD146High and CD146Low eMSC-EVs (n = 2) and then processed using a StepOne Real-time thermocycler (Applied Biosystems, LLC, Waltham, MA, USA). The analysis was performed using Qiagen’s online Analysis System (https://geneglobe.qiagen.com/us/analyze (accessed on 2 May 2025)). Mean values were normalized with SNORA73A (a small nucleolar RNA), and expression levels were calculated using the 2^−ΔCt^ method.

A miRNet-centric network visual analytics platform (https://www.mirnet.ca/ (accessed on 2 May 2025)) was used to create lncRNA interactomes. The miRNA target gene data were collected from the well-annotated database starBase v2.0. miRNA–gene interactome network refining was performed with a 1.0 betweenness cut-off. Values (with a 35-cycle cut-off point) were represented in a topology lncRNA–miRNA interactome network using a force atlas layout and hypergeometric test algorithm.

### 3.7. Chondrocytes/Synoviocytes Co-Culture Assay

Primary human chondrocytes (HCHs) were purchased and cultured in chondrocyte growth medium (PromoCell, Heidelberg, Germany) at 37 °C, 5% (*v*/*v*) CO_2_ until 80% confluent. Passage 2 synoviocytes were expanded in synoviocyte medium (ScienCell, Carlsbad, CA, USA). Chondrocyte/synoviocyte/eMSC-EV co-cultures were performed using CD146High and CD146Low eMSC-EVs for each sample (n = 2) at a concentration corresponding to EVs secreted from 1 × 10^6^ eMSCs. Cocultures were fed synoviocyte medium supplemented with a TIC inflammatory/fibrotic cocktail (15 ng/mL TNFα, 10 ng/mL IFNγ, 10 ng/mL CTGF) for 72 h.

After 72 h, chondrocytes were harvested for molecular profiling. RNA extraction was performed using the RNeasy Mini Kit (Qiagen, Frederick, MD, USA) according to the manufacturer’s instructions. Total RNA (1 μg) was used for reverse transcription with a SuperScript™ VILO™ cDNA synthesis kit (Invitrogen). A predesigned 88-gene human osteoarthritis array (GeneQuery™ Human Osteoarthritis and Cartilage repair qPCR Array Kit, ScienCell) was used with 1000 ng cDNA per culture and processed on a StepOne real-time thermocycler (Applied Biosystems, LLC). Mean values were normalized to the ACTB housekeeping gene; expression levels were calculated using the 2^−ΔCt^ method with a 34-cycle cutoff. Values were represented in a stacked bar plot as the relative-fold change in the chondrocytes with CD146High or CD146Low eMSC-EVs to the chondrocytes without eMSC-EV treatment (reference sample, 2^−ΔCt^ = X sample/X reference sample).

The functional enrichment analysis was performed using g:Profiler (version e113_eg59_p19_f6a03c19) with the g:SCS multiple testing correction method, applying a significance threshold of 0.05. The colors for different evidence codes and for log scale are described in Supplementary Table S1.

### 3.8. Angiogenesis Assay

The pro-angiogenic potential of EVs derived from CD146High and CD146Low eMSCs was evaluated by assessing their effect on endothelial wound closure in an in vitro scratch assay performed under inflammatory conditions. Human umbilical vein endothelial cells (HUVECs) were seeded at a density of 5 × 10^5^ cells per well in 6-well plates and cultured in Endothelial Cell Growth Medium (PromoCell) supplemented with Supplement Mix (PromoCell). Cells were maintained at 37 °C in a humidified 5% CO_2_ incubator until a confluent monolayer formed, typically within three days. All experimental groups were tested in both biological and technical duplicates.

At confluence, a linear scratch was introduced at the center of each well using a sterile 1000 µL pipette tip. To remove cellular debris, wells were washed twice with DPBS. The culture medium was then replaced with EV-depleted medium supplemented with pro-inflammatory cytokines: TNF-α (15 ng/mL), IFN-γ (10 ng/mL), and CTGF (10 ng/mL). Three treatment conditions were tested: no EVs (control), CD146High EVs, and CD146Low EVs, with EV doses corresponding to the secretome of 1 × 10^6^ parental cells per well.

Immediately after scratch formation (0 h), images were acquired using a phase-contrast inverted microscope (Leica) at 4× magnification. The same field of view was captured at each time point, and additional images were taken every 24 h to monitor the progression of wound closure. Image analysis was conducted using Fiji/ImageJ 2.14.0 software (ImageJ 1.54p, NIH), where the scratch area at each time point was traced and measured. Quantitative data were plotted and analyzed using GraphPad Prism (v10.4.2). Results represent the mean from replicate wells.

After 72 h, endothelial cells were harvested for molecular profiling. RNA extraction was performed using the RNeasy Mini Kit (Qiagen, Frederick, MD, USA) according to the manufacturer’s instructions. Total RNA (1 μg) was used for reverse transcription with a SuperScript™ VILO™ cDNA synthesis kit (Invitrogen). A predesigned 81-gene human endothelial cell biology array (RT^2^ Profiler™ PCR Array Human Endothelial Cell Biology, Qiagen) was obtained using 1000 ng cDNA per culture and processed using a StepOne real-time thermocycler (Applied Biosystems, LLC). Mean values were normalized to the ACTB housekeeping gene; expression levels were calculated using the 2^−ΔCt^ method with a 34-cycle cutoff. Values were represented in a stacked bar plot as the relative-fold change in the endothelial cells with CD146High or CD146Low eMSC-EVs to the endothelial cells without eMSC-EV treatment (reference sample, 2^−ΔCt^ = X sample/X reference sample).

## 4. Conclusions

In summary, eMSC-EVs carry chondroprotective and pro-angiogenic lncRNA profiles that modulate chondrocyte and endothelial cell functionality under inflammatory conditions in vitro. Notably, CD146High eMSC-EVs exhibit a superior lncRNA profile, with stronger protective effects than CD146Low eMSC-EVs counterparts. On this basis, our results reveal the potential of cell-free CD146High eMSC-EV therapeutics and warrant further investigation in animal models of inflammatory joint diseases, including OA.

## Data Availability

The original contributions presented in this study are included in the article/Supplementary Materials. Further inquiries can be directed to the corresponding author.

## Supplementary Materials

The following supporting information can be downloaded at: https://www.mdpi.com/article/10.3390/ijms262110567/s1.
