# Supplementary figures and images for: Characterizing the Long Non-Coding RNA Profile of Endometrial Mesenchymal Stem/Stromal Cell-Derived Extracellular Vesicles and Their Anti-Inflammatory Role in Osteoarthritis
Source: Int J Mol Sci. 2025 Oct 30;26(21):10567. doi: 10.3390/ijms262110567 (PMC12609597; doi:10.3390/ijms262110567)

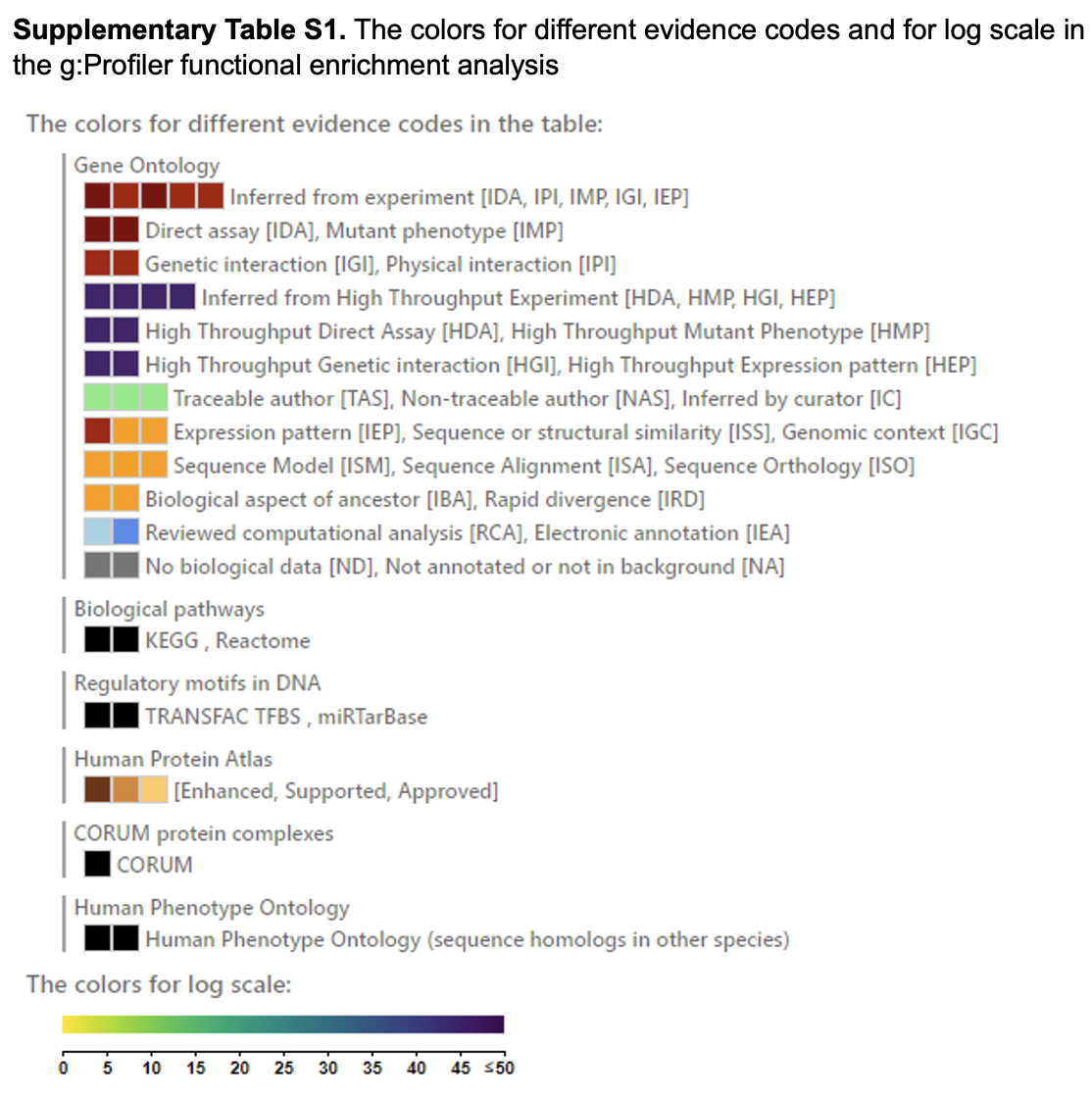

Supplement: Supplementary file 1 [file ijms-26-10567-s001.zip › eMSC Sup. Table 1.png]
